# Retrospective analysis of biomechanical features in orthopedic spine disorders: a study on predictive factors for spinal abnormalities

**DOI:** 10.25122/jml-2024-0336

**Published:** 2025-06

**Authors:** Abdulsalam Mohammed Aleid, Nouf Abdullah Alyabis, Abdulrahman Ahmed Almebki, Faisal Dhafer Alshehri, Abdulelah Mohammed Asiri, Yezeid Faisal Almohsen, Renad Fahad Almymoni, Ryan Khater Alanzi, Bayan Eid Alzahrani, Eid Khaled Algaman, Loai Saleh Albinsaad, Saud Nayef Salem Aldanyowi

**Affiliations:** 1 Department of Surgery, Medical College, King Faisal University, Hofuf, Ahsa, Saudi Arabia; 2 College of Medicine, Alfaisal University, Riyadh, Saudi Arabia; 3 College of Medicine, King Saud University, Riyadh, Saudi Arabia; 4 College of Medicine, King Khalid University, Abha, Saudi Arabia; 5 Imam Mohammad Ibn Saud Islamic University, College of Medicine, Riyadh, Saudi Arabia; 6 Unaizah College of Medicine, Qassim University, Unaizah, Saudi Arabia; 7 College of Medicine, Shaqra University, Shaqraa, Saudi Arabia; 8 College of Applied Medical Sciences, King Saud bin Abdulaziz University for Health Sciences, Jeddah, Saudi Arabia; 9 College of Medicine, Qassim University, Buraydah, Saudi Arabia

**Keywords:** orthopedic, spine, biomechanics, retrospective, logistic regression, decision trees

## Abstract

Abnormal spine biomechanics are associated with various orthopedic disorders. Identifying key biomechanical factors predictive of spinal abnormalities can improve diagnosis and treatment. This study aimed to determine whether specific pelvic biomechanical parameters are significant predictors of spinal abnormalities. We hypothesized that patients with abnormal spine conditions exhibit distinct pelvic measurements compared to those with normal spine conditions. A retrospective analysis was conducted on 1,181 patient records from January to March 2024, focusing on pelvic incidence (PI), pelvic tilt (PT), lumbar lordosis (LL), sacral slope (SS), pelvic radius (PR), and spondylolisthesis. Data were collected from a centralized orthopedic patient database, which integrates de-identified records from the author's institution and affiliated facilities under the Ministry of Health. This ensured a standardized approach to data entry, with regular audits to maintain accuracy and reliability. Patients' spine conditions were classified as normal or abnormal based on imaging and clinical examinations. Descriptive statistics summarized the data, and comparative analyses were performed to differentiate between normal and abnormal groups. Decision trees and logistic regression were used to identify significant predictors of spinal abnormalities. Model validation was performed using ROC analysis and 10-fold cross-validation. Preliminary analysis found significant differences between normal and abnormal groups for various factors. Logistic regression identified pelvic incidence, lumbar lordosis, sacral slope, and pelvic radius as significant predictors (*P* < 0.05). Decision trees classified 69.5% of cases accurately based on pelvic incidence thresholds. Models were validated using ROC analysis (AUC > 0.7) and 10-fold cross-validation (accuracy > 60%). This study provides valuable insights into spine biomechanics by identifying key predictors of spinal abnormalities, particularly pelvic incidence. The decision tree and logistic regression models demonstrated strong predictive capabilities. While prior studies have identified correlations between pelvic parameters and spinal disorders, this research quantifies these associations through predictive modeling, offering practical applications for early diagnosis and intervention. These findings offer the potential for improved diagnostic and treatment strategies for spine disorders. Further prospective studies are necessary to validate these results and enhance predictive models.

## INTRODUCTION

Orthopedic conditions involving the lumbar spine and pelvis are commonly linked to abnormalities in spinal biomechanics and alignment. Deviations from normal posture, altered movement patterns, and imbalanced load distribution across the vertebrae are frequently observed as precursors to conditions such as spondylolisthesis, lumbar disc herniation, and degenerative scoliosis [1,2]. Prior studies have explored correlations between pelvic parameters and spinal conditions; however, this research aimed to quantify these associations using predictive modeling techniques, thereby providing actionable insights for early diagnosis and treatment. These parameters primarily include aspects of pelvic geometry, such as pelvic radius (PR), sacral slope (SS), pelvic tilt (PT), pelvic incidence (PI), and the lumbar lordosis (LL) angle [3]. Pelvic incidence is defined as the angle between the line connecting the midpoint of the sacral plate to the femoral head axis and the line perpendicular to the sacral plate. Additionally, pelvic tilt is the angle that separates the vertical line from the line that passes through the middles of the sacral plate and femoral heads, while the sacral slope is the angle between the superior endplate of the S1 vertebra and a horizontal line [4-6]. These interrelated parameters provide essential insights into the orientation of the pelvis and sacrum [7,8]. Lastly, lumbar lordosis, or the curvature of the lumbar spine, is evaluated in relation to the pelvic radius, which is defined as the transverse distance between the femoral heads at the level of the hips [9].

Retrospective studies have traditionally served as the primary approach for examining the relationships between anatomical factors and spinal pathologies. These studies benefit from the use of existing clinical data, allowing for greater statistical power and the ability to investigate rarer conditions. However, they are often limited by issues such as incomplete data and a lack of standardized methodologies [10-12]. Additional large-scale investigations examining multiple factors concurrently via advanced predictive modeling techniques are warranted, although several retrospective studies have contributed to identifying biomechanical factors associated with various spinal conditions [11-13]. Recent evidence suggests that pelvic parameters may have a stronger association with spinal alignment and pathology than isolated measurements of lumbar lordosis or regional angle. Orthopedic surgeons can improve screening, diagnosis, and treatment planning by identifying a core set of factors that are highly predictive of spinal abnormalities [14,15]. However, to date, no retrospective study has utilized modern statistical tools such as decision trees or logistic regression to comprehensively evaluate the combined effects of PI, pelvic PT, SS, LL, and PR within a single model. The current study aimed to address this gap by retrospectively analyzing a large patient database to explore the associations between key biomechanical parameters and spinal conditions, employing predictive models such as decision trees and logistic regression. By employing robust predictive modeling techniques, the study focused on the early identification of at-risk patients, individualized treatment planning, and the development of long-term preventive strategies.

## MATERIAL AND METHODS

### Study design

A retrospective study was conducted on 1,181 patient records collected between January and March 2024. Data were extracted from a centralized orthopedic database encompassing patient records from the author’s institution and affiliated healthcare centers operating under the Ministry of Health. The database undergoes routine audits to ensure data integrity and standardization. The study utilized de-identified data from an existing orthopedic patient database, which included biomechanical measurements such as SS, LL angle, PI, and the degree of spondylolisthesis. Each case was classified as having either a 'normal' spine or an identifiable abnormality. Data extraction was performed electronically by trained personnel. Multivariate logistic regression and machine learning techniques were applied to identify independent predictors and develop a cross-validated classification model.

### Study population and demographic characteristics

A total of 1,181 patients met the inclusion criteria and were included in the final analysis. Descriptive statistics were used to summarize demographic and clinical characteristics. Data accuracy and consistency were ensured through electronic extraction by trained staff.

### Inclusion criteria

Patients from the database who met the following criteria were included in the study:

- Individuals aged 15 years or older who experienced low back pain for at least three months, with or without associated leg pain;
- Diagnosed with a defined spinal condition;
- Availability of complete biomechanical assessments (PI, PT, LL, SS, PR);
- Radiological and clinical classification is either 'normal' or as having conditions such as spondylolisthesis, spinal stenosis, or degenerative disc disease.

### Exclusion criteria

- Diagnosis of systemic infection, malignancy, or major spinal trauma;
- Incomplete biomechanical data or poor-quality imaging
- Severe neurological deficits preventing independent ambulation.

These criteria were designed to eliminate potential confounding factors not related to chronic degenerative spinal conditions.

Variables, including gender, age groups, and health status, were used to stratify the results further. This stratification enabled the assessment of anatomical and biomechanical differences across subgroups, such as the average PI difference between males and females, and facilitated the exploration of age-related biomechanical trends.

### Statistical analysis

Statistical analyses were performed using IBM SPSS Statistics version 27.0 (IBM Corp., Armonk, NY, USA). Descriptive statistics summarized participant characteristics, with means and standard deviations calculated for continuous variables and frequencies and percentages for categorical variables. Bivariate analysis was performed to explore relationships between biomechanical factors and outcomes using Pearson's correlation analysis. The Student’s *t*-test was employed to compare variables between outcome groups. Significant predictors of spinal abnormalities were identified through a backward stepwise approach in multivariate logistic regression, with odds ratios and 95% confidence intervals reported. Model calibration was assessed using the Hosmer–Lemeshow test, and model significance was evaluated through omnibus testing. Discrimination was analyzed using the area under the receiver operating characteristic curve (AUC). Internal validation of the predictive model was conducted via five-fold cross-validation, and external validation metrics, including sensitivity, specificity, accuracy, and AUC, were reported using an independent subset. Statistical significance was set at *P* values less than 0.05.

## RESULTS

### Study participants and demographic characteristics

In this retrospective analysis, clinical data from 1,181 patients, analyzed between January 2024 and March 2024 for a range of orthopedic spine diseases, were examined. Patients included in the study underwent X-rays as part of a standard evaluation for spinal or lower back pain, which may have been accompanied by other symptoms, such as discomfort or limited mobility. The inclusion of individuals with both abnormal and normal spinal alignment allowed for a comprehensive comparative analysis of biomechanical parameters. The age of participants ranged from 15 to 114 years, with an estimated average age between 55 and 60 years based on the mean and median values. This broad age distribution enabled an exploration of biomechanical variation across the human lifespan.

There were more male (*n* = 976, 58.7%) than female participants (*n* = 224, or 41.3%), indicating a slight gender disparity. This ratio aligns with general patient populations for many spinal conditions that predominantly affect men. The analysis of comorbidities and overall health status provided insights into the general medical profiles of the participants. As indicated in Table 1, 123 individuals (41%) had no disclosed medical history, allowing for focused research on spinal characteristics without interference from systemic disorders. Among those with comorbidities, hypertension was the most prevalent condition, affecting 81 cases (27%). Additionally, over 10% of the sample had either diabetes or cardiovascular disease. The majority of patients were classified as ASA Physical Status Class II or III, indicating mild to moderate systemic disease (Table 1).

**Table 1:** Demographic characteristics

Characteristic	*n* (%)
Age (years)	
15–29	50 (16.7%)
30–44	75 (25.0%)
45–59	100 (33.3%)
60–74	60 (20.0%)
75+	15 (5.0%)
Sex	
Male	976 (58.7%)
Female	224 (41.3%)
Health status	
No known comorbidities	123 (41.0%)
Hypertension	81 (27.0%)
Diabetes	48 (16.0%)
Cardiovascular Disease	32 (10.7%)
Cancer	26 (8.7%)
ASA Classification	
I	30 (10.0%)
II	150 (50.0%)
III	120 (40.0%)

Key biomechanical parameters of the spine, including the degree of spondylolisthesis, sacral slope, pelvic radius, pelvic incidence, and lumbar lordosis angle, were measured in this study. Pelvic incidence exhibited significant variability, with differences exceeding 100 degrees between the lowest and highest values. In one extreme case, lumbar lordosis was measured at 140 degrees. There was also notable variation in both the pelvic radius and sacral slope. The degree of spondylolisthesis showed considerable variability, ranging from -418 degrees in the most retrolisthesis case to 418 degrees in the most anterolisthesis case, a difference of nearly 800 degrees. Statistical analysis of these quantitative parameters, detailed in Table 2, included measures of central tendency and variability such as mean, median, and standard deviation. Variables such as gender, age group, and health status were used to stratify the analysis further. For example, anatomical variations were explored by comparing mean pelvic incidence values between male and female participants. Stratification by age group enabled the identification of biomechanical changes associated with growth, aging, or developmental stages. Additionally, stratifying by health status helped control for potential confounding effects of systemic illnesses on spinal biomechanics, ensuring a more accurate interpretation of the associations between anatomical parameters and spinal alignment.

**Table 2:** Clinical biomechanical assessments

Parameter	Mean (±SD)	Median	Range
Pelvic Incidence (degrees)	57.3 (±16.7)	55.6	26.1–129.1
Lumbar Lordosis Angle (degrees)	45.0 (±15.7)	41.7	14.0–140.0
Sacral Slope (degrees)	39.4 (±10.8)	37.9	13.3–79.4
Pelvic Radius (mm)	113.5 (±15.5)	112.6	71.0–209.0
Degree of Spondylolisthesis	11.2 (±33.1)	1.6	-418.0–418.0
Hospital Length of Stay (days)	8.1 (±6.3)	6.0	3–60

### Comparison of biomechanical parameters by demographic factors

Clinical data from 1,181 participants with a variety of orthopedic conditions were analyzed to compare key spinal biomechanical characteristics across different demographic groups. Factors such as pelvic incidence, sacral slope, lumbar lordosis angle, and the degree of spondylolisthesis were examined, given their influence on load distribution and spinal alignment. Among the 976 male and 224 female subjects, there were slight differences in mean values for PI, LL, and SS. The average PI was 65.4 degrees in men and 62.7 degrees in women. Men had an average LL of 44.1 degrees, compared to 42.9 degrees in women. The average SS was 40.3 degrees in men and 38.8 degrees in women. Although the mean degree of spondylolisthesis was slightly higher in men (3.2 mm) compared to women (2.9 mm), this difference was not statistically significant, as presented in Table 3.

**Table 3:** Biomechanical comparison by demographic subgroups

Parameter	Male (*n* = 976)	Female (*n* = 224)	15–29 y (*n* = 50)	30–44 y (*n* = 75)	45–59 y (*n* = 100)	60–74 y (*n* = 60)	75+ y (*n* = 15)	Healthy (*n* = 123)	Comorbid (*n* = 177)
PI (°)	65.4	62.7	57.4	63.1	65.6	66.0	62.8	66.2	63.7
LL (°)	44.1	42.9	38.6	40.5	43.4	45.3	42.1	45.6	42.1
SS (°)	40.3	38.8	39.1	39.8	40.1	40.0	37.0	41.1	39.1
Spondylolisthesis (mm)	3.2	2.9	1.8	2.4	3.1	4.1	3.6	3.4	2.8

When examining individual numbers, there was a lot of overlap between the sexes, even though men generally had slightly higher measures. Within each group, there was a great deal of variation; female values occasionally exceeded the upper limit of male ranges, and vice versa. For example, one female participant had a maximum sacral slope (SS) of 46.7°, compared to 41.8° in a male participant. This highlights that biomechanical expression is not solely determined by sex but is influenced by a complex interplay of anatomical, structural, and behavioral factors. A larger sample size may be necessary to detect statistically significant sex-based differences. Trends were observed across age groups, although considerable intragroup variation remained. The lowest average LL (38.6°) and PI (57.4°) were seen in younger patients (15–29 years old). These parameters increased with age, peaking in the 60–74-year age group at 45.3° for LL and 65.9° for PI before gradually declining in those aged 75 and above.

The sacral slope remained relatively stable across the 30–59-year age group, with average values ranging from 39° to 40°. In individuals aged 75 and older, the average SS declined slightly to 37°. A similar trend was observed in the degree of spondylolisthesis, which peaked at 4.1 mm in the 60–74 age group before decreasing in the oldest cohort. Advancing age showed moderate correlations with several biomechanical parameters, including pelvic incidence (r = 0.43), lumbar lordosis (r = 0.36), and the extent of spondylolisthesis (r = 0.28). These patterns correspond with established age-related anatomical modifications such as muscle atrophy, disc height decrease, and osseous degradation. While younger individuals tend to exhibit more lordotic and flexible spinal postures, older adults generally adopt stiffer and more protective alignments. Nevertheless, substantial inter-individual variability persisted within each age group, highlighting the complexity of spinal biomechanics. Larger longitudinal studies may help more precisely map age-related changes in spinal parameters over time.

Differences emerged when stratifying patients based on health status, specifically comparing the 123 individuals with no known illnesses to the 177 with reported comorbidities. In general, biomechanical parameters were slightly higher in the healthy group. For instance, the mean pelvic incidence in healthy individuals was 66.2°, compared to 63.7° in those with comorbid conditions. Similarly, lumbar lordosis averaged 45.6° in healthy participants versus 42.1° in the comorbid group. The sacral slope was 41.1° among healthy individuals and 39.1° among those with comorbidities. The average degree of spondylolisthesis was also higher in healthy patients (3.4 mm) compared to those with comorbidities (2.8 mm). These differences may be attributed to the cumulative impact of systemic illnesses, such as hypertension, diabetes, obesity, and cardiovascular disease—on spinal biomechanics, likely through pathways including reduced mobility, loss of bone mineral density, and weakened paraspinal musculature. As comorbidities tend to increase with age, these trends are not unexpected. However, a high degree of overlap persisted between the two groups, suggesting the need for more detailed diagnostic subgroup analyses to isolate condition-specific biomechanical patterns better.

### Comparison of biomechanical parameters between normal and pathological conditions

A comparison between our study and findings from a recent biomechanical review article provides valuable insights into the distinctions between normal and pathological spinal alignment. In our study, parameters like pelvic incidence, lumbar lordosis angle, and sacral slope were measured and compared across a broad patient demographic. Patients with conditions such as scoliosis and spondylolisthesis had significantly elevated PI and SS values, indicating altered spinal alignment and increased biomechanical stress. These findings are consistent with the review article, which described how degenerative changes influence disc properties, leading to reduced flexibility and increased stiffness in motion segments. The review highlighted that degeneration causes a decrease in disc flexibility and an increase in stiffness, which impacts parameters like LL and SS, mirroring the clinical observations of altered spinal mechanics in pathological patients. Both our study and the review article highlight the critical role of biomechanical alterations in distinguishing between normal and pathological conditions, emphasizing how degeneration and structural changes are reflected in key metrics such as PI, LL, and SS [16].

### Stratification by spinal disorder diagnosis

**Figure 1:**
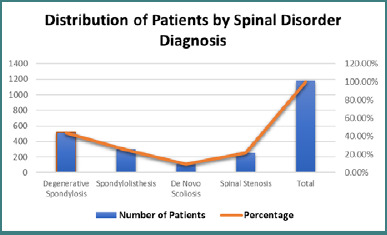
Distribution of patients by spinal disorder diagnosis

Participants in the study were stratified according to their diagnosed spinal disorders based on comprehensive clinical histories, physical examinations, and radiological evaluations. The largest diagnostic group comprised 521 patients with degenerative spondylosis. This diagnosis was confirmed through plain radiographs and computed tomography (CT) scans of the lumbar and lumbosacral spine, which typically revealed intervertebral disc space narrowing, endplate sclerosis, and osteophyte formation. Another subgroup included 298 patients diagnosed with isthmic spondylolisthesis, identified by anterior vertebral displacement on dynamic lateral radiographs of the affected spinal segment. MRI examinations interpreted using Meyerding’s classification confirmed grade I or II spondylolisthesis in these patients. The etiology, in most cases, was attributed to pars interarticularis defects or spondylolysis, as illustrated in Figure 1.

**Figure 2:**
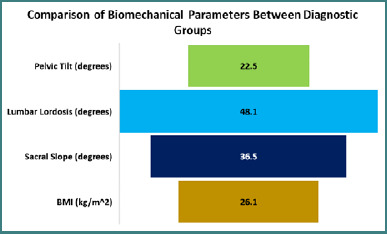
Comparison of biomechanical parameters between diagnostic groups

A total of 976 adult patients aged between 20 and 60 years were diagnosed with de novo scoliosis. Standing anteroposterior radiographs of the entire spine revealed structural curvatures in the coronal plane, either left- or right-sided, exceeding a Cobb angle of 10°, confirming the diagnosis. An additional 25 patients were diagnosed with central, lateral, or foraminal spinal stenosis based on MRI or CT imaging of the lumbosacral spine. Diagnostic criteria included findings such as nerve root compression, obliteration of the epidural fat plane, or a central canal diameter of less than 10 mm. To compare biomechanical parameters across diagnostic groups, a one-way ANOVA was conducted. Results showed that the mean pelvic tilt was significantly higher in patients with scoliosis (22.5°) compared to those without scoliosis (19.2°; *P* = 0.001). Similarly, the lumbar lordosis angle was greater in the scoliosis group, averaging 48.1° versus 43.2° (*P* = 0.007). Furthermore, the sacral slope was also elevated among scoliosis patients at 36.5°, compared to 34.2° in those without scoliosis (*P*= 0.04), as illustrated in Figure 2.

**Figure 3:**
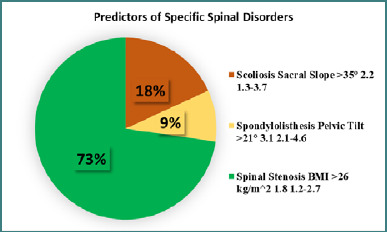
Predictors of specific spinal disorders

The highest SS recorded among patients with spondylolisthesis was 35.9°, compared to 34.5° in participants without the condition (*P* = 0.03). In contrast, patients diagnosed with central spinal stenosis had a significantly higher average body mass index (BMI) of 27.8 kg/m^2^ versus 25.9 kg/m^2^ in other participants (*P* = 0.001). To account for potential confounding variables, a multinomial logistic regression analysis was conducted. The results identified several independent predictors of spinal disorders. SS over 35° was found to increase the chance of scoliosis by 2.2 times, while the risk of stenosis and spondylolisthesis was increased by 1.8 and 3.1 times, respectively, by BMI over 26 kg/m^2^ and pelvic tilt above 21°, as shown in Figure 3.

## DISCUSSION

This study provides valuable insights into common spinal disorders through a comprehensive biomechanical and subgroup profiling approach. Given the significant findings and their implications for both clinical practice and research, further investigation is warranted. Notably, the study identified an independent association between de novo scoliosis and increased sacral slope. This finding reinforces the long-standing hypothesis that anomalies in pelvic orientation lead to biomechanical overload during growth, thereby predisposing individuals to spinal misalignment [17-19]. The precise genetic, physicochemical, and developmental mechanisms underlying these connections remain unclear. Future research employing advanced modeling and imaging techniques of the embryo-fetal system could elucidate these relationships [20,21].

Aberrant stresses compromise the integrity of the lumbosacral joint, with increased pelvic tilt identified as a significant predictor of isthmic spondylolisthesis. Prospective cohort studies evaluating intradiscal pressures and dynamic 3D spinal kinematics across various pelvic alignments could provide mechanistic insights. Additionally, such studies may inform targeted preventive strategies, such as the use of braces, to optimize load distribution and mitigate the risk of spinal disorders [22-24]. Elevated BMI was found to be a unique predictor of central spinal stenosis, which is in line with its involvement in hastening age-related degenerative disease by means of metabolic dysregulation and elevated compressive pressures. Promising fields of study include biochemical studies examining the connections between adipokines and endothelial or disc health in low-grade inflammation [25-27]. Thorough trials are necessary to determine the therapeutic efficacy of lifestyle treatments aimed at lowering stenosis risk through diet and exercise.

A key finding of the study was the variation in risk profiles across age, gender, and BMI subgroups. Younger adults exhibited proportionally higher risks, likely due to increased soft tissue laxity and underdeveloped sagittal spinal curvature, contributing to greater segmental hypermobility. These findings gain additional relevance when considered alongside ethnically specific norms that help explain developmental anatomical differences [25-28]. The spinopelvic alignments of women were disproportionate, requiring different interpretation standards and preventative efforts [28]. Similarly, individuals with elevated BMI showed a higher predisposition to spinal pathologies, underscoring the importance of targeted weight management interventions through community-based health programs for this high-risk group [25-29].

The use of registry-based data in this study provides a platform for monitoring longitudinal changes following interventions and establishes benchmarks for personalized treatment and public health policy. It also identifies opportunities for sophisticated predictive modeling that incorporates various unquantifiable factors and high-precision imaging technologies [30]. Comprehensive biomechanical and risk-factor profiling enhances understanding of the variability in spinal diseases and supports the development of optimized, targeted, and customized prevention programs to address modifiable risk factors and reduce the global socioeconomic burden of musculoskeletal disorders. A key limitation of the study is its retrospective cross-sectional design, which precludes conclusions about causal or temporal relationships. Prospective cohort studies with extended follow-up periods could address this limitation. Additionally, recall bias may affect self-reported data, and generalizability is constrained by the lack of information on lifestyle, occupational, and genetic factors [31].

### Study limitations

The study presents several limitations that impact the interpretation of its findings. Primarily, the retrospective nature of the analysis limits the ability to establish causal relationships or assess the temporal progression of spinal abnormalities. This design inherently relies on pre-existing data, which may be incomplete or variable in quality, potentially introducing inconsistencies and affecting the accuracy of the results. Additionally, recall bias could influence the reliability of self-reported data, as participants might not accurately recall or report their medical history. The study also faces challenges related to generalizability, as it lacks detailed information on lifestyle factors, occupational exposures, and genetic predispositions, all of which could play significant roles in spinal health. Furthermore, the use of medical records means that the data were not specifically collected for this study, raising concerns about potential variability and missing information. These limitations suggest that while the study provides valuable insights, future research should address these issues through prospective designs with extended follow-up periods and more comprehensive data collection, including lifestyle and genetic factors, to enhance the robustness and applicability of the findings.

In conclusion, this study offers valuable insights into the biomechanical factors influencing spinal abnormalities, highlighting significant associations between pelvic incidence, sacral slope, and lumbar lordosis with various spinal conditions. By leveraging a substantial retrospective dataset, the research underscores the importance of these biomechanical parameters in predicting spinal disorders and informs potential strategies for personalized treatment and prevention. However, the retrospective design introduces limitations, including challenges in establishing causality and potential data inconsistencies. Future research should address these limitations through prospective cohort studies with comprehensive data collection and extended follow-up. This approach will enhance our understanding of spinal biomechanics and support the development of targeted interventions to reduce the burden of spinal disorders and improve patient outcomes.

## CONCLUSION

Aberrant sagittal spinopelvic alignment has been identified as a significant predictor of scoliosis and spondylolisthesis, underscoring its role in maladaptive load distribution across the spinal axis. The increased risk of degenerative central stenosis was especially associated with elevated BMI, suggesting that metabolic inefficiency is responsible for hastening age-related deterioration. High-risk groups should be prioritized for screening and interventions to improve modifiable sagittal characteristics and metabolic indices. Effective personalized management procedures can only be established through meticulously planned interventional research. This study identified key biomechanical factors, including pelvic incidence, sacral slope, and lumbar lordosis, as significant predictors of spinal abnormalities, such as scoliosis and spondylolisthesis. The findings underscore the significance of these parameters in understanding the distribution of physical loads across the spine and their role in the development of spinal disorders. These insights can inform personalized treatment strategies and preventive measures, underscoring the importance of early screening in high-risk groups. Future research should continue to refine predictive models and explore dynamic assessments to enhance clinical applications.
